# Biofilm-specific uptake of a 4-pyridone-based iron chelator by *Pseudomonas aeruginosa*

**DOI:** 10.1007/s10534-020-00281-x

**Published:** 2021-01-11

**Authors:** Sharareh Houshmandyar, Ian M. Eggleston, Albert Bolhuis

**Affiliations:** Department of Pharmacy and Pharmacology, University of Bath, Bath, BA2 7AY UK

**Keywords:** CP94, Deferiprone (DFP), Iron chelator, *Pseudomonas aeruginosa*, Biofilm

## Abstract

Iron is an essential nutrient for virtually all microbes and limiting the concentration of available iron is a potential strategy to be used as an alternative to antibiotic treatment. In this study we analysed the antimicrobial activity of two chelators, specifically 3-hydroxy-1,2-dimethyl-4(1H)-pyridone (deferiprone, DFP), which is clinically approved for the treatment of iron overload disorders, and its 1,2-diethyl homologue, CP94. Both compounds showed moderate activity towards planktonically growing *P. aeruginosa* cells, and the mechanism of action of these chelators was indeed by limiting the amount of free iron. Surprisingly, the compounds behaved very differently when the cells were grown in biofilms. DFP also showed inhibitory effects on biofilm formation but in contrast, CP94 stimulated this process, in particular at high concentrations. We hypothesised that CP94 behaves as an iron carrier, which was confirmed by our observation that it had antimicrobial synergy with the toxic metals, gallium and copper. This suggests that *P. aeruginosa* produces a biofilm-specific transport protein that recognises CP94 but not the closely related compound DFP.

**Supplementary Information:**

The online version contains supplementary material available at 10.1007/s10534-020-00281-x.

## Introduction

Iron is essential for virtually all living organisms as it is required in many fundamental cellular processes such as respiration and nucleic acid metabolism (Daher et al. 2017). In the human body, most iron is not freely available as it is bound to hemoproteins and other iron-binding proteins such as transferrin, ferritin and lactoferrin. As a result, the concentration of free iron is very low, which has several important consequences. Firstly, iron can be toxic as, due to its redox potential, it has the capacity to lose and gain electrons in a biological environment leading to the generation of reactive oxygen species (ROS). Limiting the labile iron pool thus reduces the potential for iron-mediated oxidative damage to tissues (Cabantchik 2014). Secondly, low levels of labile iron ensure that this nutrient is not readily available to pathogenic microorganisms, which require iron for many processes including virulence, infectivity and biofilm formation (Begg 2019). To overcome this, bacteria have developed several mechanisms to acquire iron from the host, by for instance the secretion of siderophores, which are low molecular weight compounds with high affinity for ferric iron and which sequester and transport ferric iron into bacterial cells (Hider and Kong 2010; Neilands 1995).

As iron is such an important nutrient for pathogens, a number of studies have attempted to use iron chelators as antimicrobial agents, either as an alternative or addition to antibiotic treatment. Indeed, several chelators have been shown to have antimicrobial activity either alone or in combination with antibiotics or antifungals. This includes two clinically-approved chelators that are used for the treatment of iron-overload diseases, namely deferoxamine (DFO) (Lowy et al. 1984; Moon et al. 2011; van Asbeck et al. 1983a) and deferiprone (DFP) (Lai et al. 2016; Thompson et al. 2012; Zarember et al. 2009). However, the literature on the activity on these chelators appears contradictory. For instance, one study showed there was no activity for DFO against a range of common bacterial pathogens (Thompson et al. 2012), whereas another study (Kim and Shin 2009) showed that its activity on staphylococci is strain- and species-dependent and sometimes even stimulatory towards bacterial growth. Furthermore, it has been shown that while DFP can be inhibitory to growth of *Pseudomonas aeruginosa*, at sub-inhibitory concentrations it can stimulate growth by acting as an iron carrier (Visca et al. 2013). It was therefore concluded that the therapeutic use of iron chelators should be carefully assessed to avoid the stimulation of growth of particular pathogens.

**Fig. 1:**
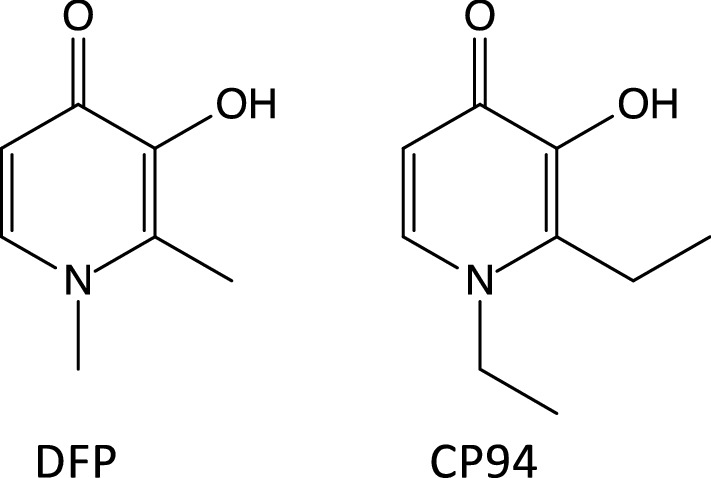
Chemical structures of the hydroxypyridone iron chelators used in this study, DFP and CP94

In this study we analysed the activity of the chelator CP94, which is a 1,2-diethyl analogue of DFP (Fig. 1). It has been shown to be more effective than DFP in removing iron in a number of mammalian model species such as rat (Singh et al. 1992), but its antimicrobial activity has not been investigated. In this study we compared the activity of both CP94 and DFP on *P. aeruginosa*, which is a common opportunistic pathogen that can cause, for instance, lung and wound infections (Mulcahy et al. 2014). The results show that both chelators have moderate antimicrobial activity against planktonic cells. Surprisingly, the chelators were shown to behave very differently when the bacteria were grown as biofilms: DFP was inhibitory at high concentrations, but CP94 stimulated biofilm formation as it was taken up selectively by *P. aeruginosa* cells in the biofilm state.

## Experimental

### Bacterial strains and growth conditions

The following *P. aeruginosa* strains were used in this study: *P. aeruginosa* PAO1 (Holloway 1955), *P. aeruginosa ΔpvdA ΔpchD* PAO1 (ΔΔPAO1) (Visca et al. 2013) and *P. aeruginosa* IST27 (Leitão et al. 1996). PAO1 is a commonly used laboratory strain, whereas IST27 is a mucoid strain isolated from a cystic fibrosis patient. Other species that were used are *Escherichia coli* BW25113 (Grenier et al. 2014) and *Salmonella enterica* serovar Typhimurium NTB6 (Kortman et al. 2015). Bacteria were grown as indicated in lysogeny broth (LB; 10 g/L tryptone, 5 g/L yeast extract, 10 g/L NaCl) or Iscove’s Modified Dulbecco’s Medium (IMDM; Sigma-Aldrich). For planktonic growth experiments, pre-cultures were grown overnight in a shaking incubator at 37 °C, diluted 100-fold in fresh medium and growth was continued at 37 °C. For biofilm experiments, IMDM was supplemented with 0.5% glucose, and the biofilms were grown as indicated below.

### Minimum inhibitory concentrations

Minimum inhibitory concentrations (MIC) values of DFP (Sigma-Aldrich) or CP94 (Chemspace) were determined using a macrobroth dilution method as described (Andrews 2001), using either LB or IMDM as the growth medium. Concentrated stock solutions were made in DMSO (CP94) or water (DFP); final concentrations of solvent were < 1%, which did not impact the outcome of the experiments. Experiments with IMDM were done in the presence of 10 µM ferric ammonium citrate where indicated.

### Biofilm assays

Biofilms were grown as previously described (Beeton et al. 2014) with minor modifications. Briefly, overnight bacterial cultures were grown in IMDM and diluted to an optical density at 600 nm (OD600) of 0.05 in fresh IMDM supplemented with 0.5% glucose. These inoculums were dispensed (200 µL) into 96-well plate wells (Costar, Corning). Afterwards, the plates were incubated for 24 h at 37 °C on an orbital rotating platform (Stuart mini gyro-rocker SSM3) at 20 rpm. Following biofilm formation, the supernatant was removed, and wells were washed three times with phosphate buffered saline (PBS; Sigma-Aldrich) followed by drying at 60 °C for 45 min. The biofilms were then stained with 0.1% w/v crystal violet for 15 min and washed three times gently by plunging in lukewarm tap water. Finally, 200 µL 30% v/v acetic acid was added to dissolve the crystal violet and the absorbance at 595 nm was measured using an automatic plate reader (BMG Labtech). Experiments were performed in the presence or absence of DFP and CP94 and, where indicated, Ga_3_(SO_4_)_3_ or CuCl_2_ (both from Sigma-Aldrich) were added. All biofilm assays were repeated at least three times independently, with 6 wells per experiment. Absorbance values were normalised to the mean of the control sample, thus expressing biofilm formation as a percentage.

### Adherence

The procedure to measure adherence of cells was similar to biofilm formation, but instead of 96-well plates, 6-well polystyrene plates were used which were inoculated with 2 mL IMDM containing a 1000-fold diluted overnight culture of *P. aeruginosa* PAO1, similar to previously published methods (Toledo-Arana et al. 2001). Briefly, the plates were incubated for 6 h, after which the plates were washed with PBS twice to remove planktonic cells, followed by drying at 60 °C and staining with 0.1% crystal violet. The plates were then washed with lukewarm tapwater as above, after which the plates were observed with light microscopy. It was verified that cells were distributed fairly evenly, and then the number of adherent cells in four randomly chosen fields of view were counted, and each experiment was repeated four times.

### Statistical analysis

Data are expressed as the mean ± standard deviation. Group means were compared using one-way ANOVA, followed by Tukey’s or Dunnett’s multiple comparison test. Differences were considered statistically significant at p values of < 0.05.

## Results

### Antimicrobial activity

The activity of DFP and CP94 against *P. aeruginosa* was determined in iron-replete and iron-poor conditions using LB and IMDM, respectively. LB is a nutrient-rich medium which contains, based on data supplied by the manufacturer of its components, 5–12 µM iron, and similar values have also been reported in the literature (Abdul-Tehrani et al. 1999; Cunrath et al. 2016; Diggle et al. 2007). In contrast, IMDM is a chemically defined tissue-culture medium that lacks iron in its formulation. It should be noted, however, that IMDM still supports the growth of *P. aeruginosa*, albeit at a slower rate as compared to LB or IMDM supplemented with iron (data not shown), and also other bacteria such as *Escherichia coli* and *Salmonella enterica* can grow in IMDM (Kortman et al. 2015). Iron is essential for growth of these bacteria, indicating that IMDM does contain the low levels of iron that are sufficient to support the growth of these bacteria.

In LB, the MIC values were 1024 (7.4 mM) and 2048 µg/mL (12 mM) for DFP and CP94, respectively, for both PAO1, a commonly used laboratory strain, and IST27, a mucoid isolate from a cystic fibrosis patient (Table 1). In iron-poor conditions, the strains were considerably more sensitive to both chelators, with MIC values being 4–eightfold lower as compared to those in LB. To test whether these lower MIC values in IMDM were due to the absence of iron only, 10 µM Fe^3+^ was added to IMDM, resulting in an iron concentration that is similar to that in LB medium. As shown in Table 1, this indeed resulted in MIC values similar to those observed in LB medium.

**Table 1:** MIC values (µg/mL) of DPF and CP94 in different media

Compound	Strain	Medium		
		LB	IMDM	IMDM + Fe^3+^
DFP	PAO1	1024	128	1024
	IST27	1024	128	1024
CP94	PAO1	2048	256	2048
	IST27	2048	512	2048

*The effect of DFP and CP94 on* P. aeruginosa *biofilm formation.*

**Fig. 2:**
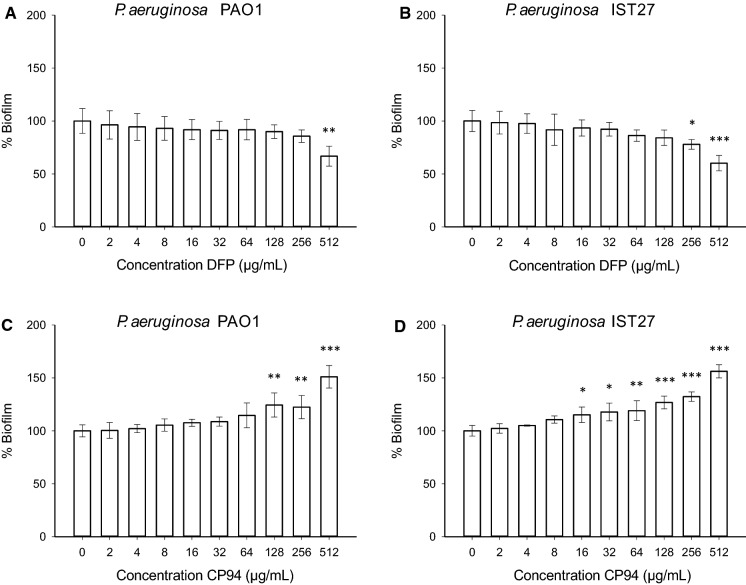
Effects of the concentration of DFP (panels **a** and **b**) and CP94 (panels **c** and **d**) on the biofilm formation in IMDM of PAO1 (panels **a** and **c**) and IST27 (panels **b** and **d**). The error bars show the standard deviation of 3–4 independent experiments. Significance was compared to the controls (0 µg/mL) using a 1-way ANOVA followed by Dunnett’s multiple comparison test (*p < 0.05; **p < 0.01; ***p < 0.001)

Next, the effect of DFP and CP94 on biofilm formation of *P. aeruginosa* was tested in IMDM. With DFP, significant reductions in biofilm formation were observed at concentrations above the MIC value, but even at the highest concentration tested (512 µg/mL; 3 mM) still 60–65% of the biofilm biomass remained (Fig. 2a, b) for both PAO1 and IST27 as compared to the biomass formed in the absence of compound.

At sub-MIC levels of DFP (64 µg/mL), planktonic growth of *P. aeruginosa* PAO1 is reduced (Supplementary Fig. 1), indicating that the reduction in biofilm formation in the presence of DFP is an effect of the reduction in the rate of growth. Planktonic growth was also reduced in the presence of sub-MIC levels of CP94 (Supplementary Fig. 1) but, surprisingly, more biofilm was formed (Fig. 2c, d), which was statistically significant at high concentrations above the MIC. With both PAO1 and IST27, 512 µg/mL CP94 resulted in an increase in biofilm formation of 50–60%. At this concentration, we also analysed the effects on the early stages of biofilm formation by counting the number of adherent cells after 6 h of incubation. In the presence of CP94, we counted > threefold the number of adherent cells as compared to the control: in the presence of CP94 there were 73 (± 23) cells per field of view, whereas there were 22 (± 7) cells per field of view in the absence of CP94; this difference was statistically significant (n = 4; Student’s t-test, p = 0.01).

*The effect of CP94 on* E. coli *BW25113 and* S. *Typhimurium NTB6.*

**Fig. 3:**
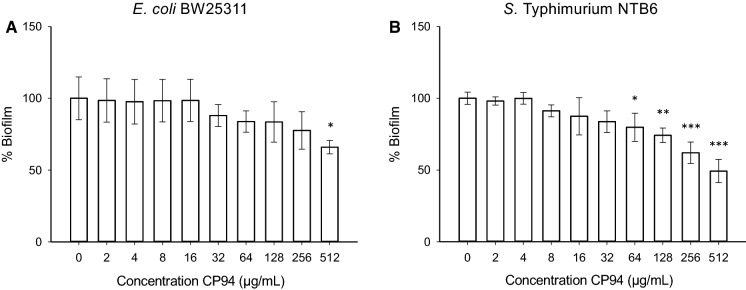
Effects of the concentration of CP94 (panels **a** and **b**) on the biofilm formation of *E. coli* BW25113 (**a**) and *S.* Typhimurium NTB6 (**b**) in IMDM. The error bars show the standard deviation of 3 independent experiments. Significance was compared to the controls (0 µg/mL) using a one-way ANOVA followed by Dunnett’s multiple comparison test (*p < 0.05; **p < 0.01; ***p < 0.001)

In view of these results, we decided to investigate whether CP94 could also potentiate the biofilm formation of two other Gram-negative bacteria, *i.e. E. coli* and *S.* Typhimurium. Interestingly, CP94 inhibited biofilm formation of both of these bacteria (Fig. 3), suggesting that the stimulatory effect of CP94 is specific to *P. aeruginosa*, in particular considering that the MIC values for CP94 against *E. coli* and *S.* Typhimurium were in the same range as for *P. aeruginosa*, with values of 128 and 512 µg/mL, respectively.

*The effect CP94 on* P. aeruginosa *PAO1* ΔpvdA ΔpchD.

**Fig. 4:**
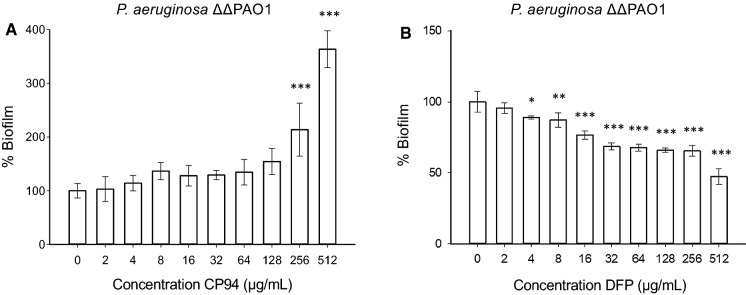
Effect of CP94 and DFP on the biofilm formation of ΔΔPAO1 in IMDM. The error bars show the standard deviation of 3 independent experiments. Significance was compared to the control (0 µg/mL) using a one-way ANOVA followed by Dunnett’s multiple comparison test (*p < 0.05; **p < 0.01; ***p < 0.001)

As shown above, the effect of DFP and CP94 is very similar towards planktonic cells, but the outcome is very different when *P. aeruginosa* is growing as biofilms in iron-poor conditions. We speculated that CP94 but not DFP might be taken up by cells in biofilms and act as an iron carrier. To investigate this in more detail, the effects of CP94 were investigated on PAO1 Δ*pvdA* Δ*pchD* (ΔΔPAO1), a mutant of PAO1 that does not produce its two endogenous iron carriers, the siderophores pyochelin and pyoverdine (Visca et al. 2013). As planktonic cells, this mutant had the same resistance to DFP as the parental strain, and was somewhat less resistant to CP94, albeit that this differed by only one doubling dilution and therefore not significant (CP94 MIC values: LB, 1024 µg/mL; IMDM, 256 µg/mL; IMDM + Fe^3+^, 1024 µg/mL). As planktonic cells, ΔΔPAO1 grows slower than the parental strain in IMDM, but biofilm formation is at similar levels (data not shown). When testing the effects of CP94 on biofilm formation, the stimulation of biofilm formation was even more pronounced as observed with PAO1 or IST27; in this case, at a concentration of 512 µg/mL the increase in biomass was 3.5-fold (Fig. 4a). The effect of DFP on ΔΔPAO1 was similar as observed for the parental strain, with a reduction in biofilm formation to approximately 50% at a concentration of 512 µg/mL (Fig. 4b).

*The combined effect of CP94 with either Ga*^*3*+^ *or Cu*^*2*+^ *on* P. aeruginosa *biofilm formation.*

**Fig. 5:**
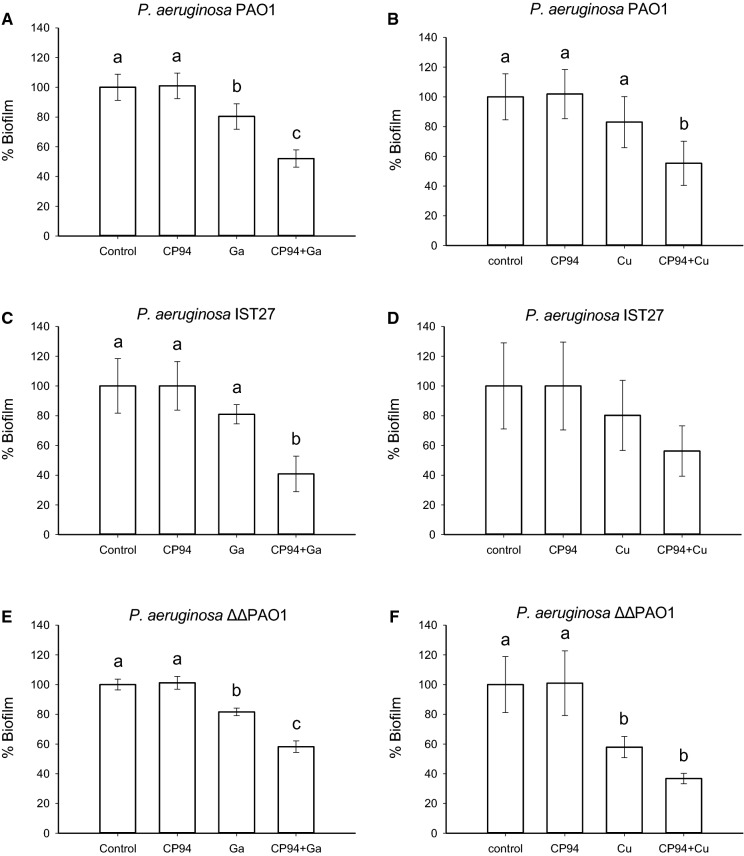
Activity of CP94 with Ga^3+^ (**a**, **c**, **e**) and Cu^2+^ (**b**, **d**, **e**) against biofilms of *P. aeruginosa* PAO1 (**a**, **b**), IST27 (**c**, **d**) and ΔΔPAO1 (**e**, **f**) in IMDM. Error bars indicate standard deviation of at least 3 independent experiments. Statistical analysis was done using a one-way ANOVA followed by a Tukey’s posthoc test to compare pairwise the means of the different treatments. Different letters indicate significant differences between treatments (p < 0.05), whereas treatments indicated with the same letter are statistically not different from each other. If no letters are shown, none of the treatments showed statistically significant differences

We hypothesised that if CP94 indeed acts as an iron carrier, it also might promote the uptake of other metals such as Ga^3+^ or Cu^2+^ and, since these are toxic to bacteria, CP94 should increase the antimicrobial activity of these metals towards *P. aeruginosa*. To test this, we firstly determined the concentrations of Ga^3+^ or Cu^2+^ salts that resulted in a moderate (20–40%) inhibition of biofilm formation in IMDM, which was 1 µM and 1 mM respectively. These concentrations were then combined with a low concentration of CP94 (2 µg/mL = 12 µM) and added to biofilms of the three *P. aeruginosa* strains used (PAO1, IST27, and ΔΔPAO1). As shown in Fig. 5, in all strains tested the biomass of biofilms treated with a CP94-Ga complex was significantly reduced when compared to treatment with CP94 or Ga^3+^ alone. In the case of copper, which by itself is less toxic than gallium, these trends were the same albeit that the differences were not always statistically significant (*e.g.* IST27 Fig. 5D). Thus, CP94 enhances the antimicrobial activity of both Ga^3+^ and Cu^2+^, but to verify that was indeed a biofilm-specific effect, the CP94-Ga complex was also tested on planktonic cells. As shown in Supplementary Fig. 2, this complex or its individual components did indeed not have an effect on the growth rate of *P. aeruginosa* PAO1 and IST27, confirming that the effects seen are biofilm-specific. We also tested whether DFP increased the antimicrobial activity of Ga^3+^, but there was no difference in the presence of DFP when combined with 1 µM Ga^3+^ (Supplementary Fig. 3).

*The combined effect of CP94 with Fe*^*3*+^*on* P. aeruginosa *biofilm formation.*

**Fig. 6:**
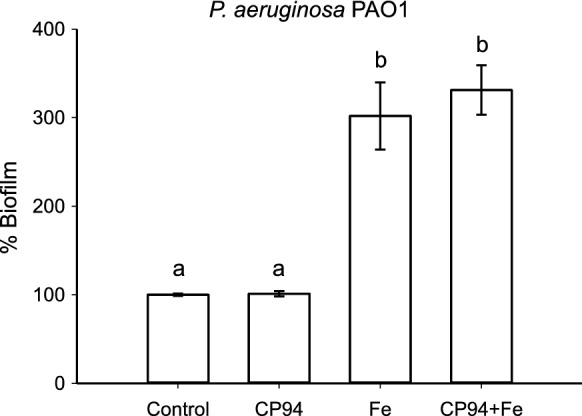
Activity of CP94 with Fe^3+^ on biofilms of *P. aeruginosa* PAO1. Error bars indicate standard deviation of at least 3 independent experiments. Statistical analysis was done using a one-way ANOVA followed by a Tukey’s posthoc test to compare pairwise the means of the different treatments. Different letters indicate significant differences between treatments (p < 0.05), whereas treatments indicated with the same letter are statistically not different from each other

Above we demonstrate that 2 µg/mL (12 µM) CP94 enhances the antimicrobial activity of toxic metals. Using the same concentration of CP94, we also analysed what the effect on biofilm formation of *P. aeruginosa* PAO1 is in the presence of CP94 saturated with Fe^3+^, and the same concentration of Fe^3+^ without CP94. As CP94 forms a 3:1 complex with Fe^3+^, the concentration of Fe^3+^ used here was 4 µM. As shown in Fig. 6, both Fe^3+^ alone and CP94 plus Fe^3+^ stimulated biofilm formation, and this was statistically significant. There was no significant difference between bioiflms grown in the presence of CP94 with Fe^3+^ or Fe^3+^ alone, indicating that at a concentration of 4 µM Fe^3+^, the transport of Fe^3+^ is not a rate limiting factor for biofilm formation. Higher concentrations of CP94, or CP94 saturated with Fe^3+^, did not further increase the amount of biofilm formed (data not shown).

## Discussion

Iron is an important nutrient for most microbes, and bacteria such as *P. aeruginosa* have therefore developed high-efficiency iron uptake systems that allow the bacteria to grow under iron-limited conditions, including when they grow as biofilms (Banin et al. 2005; Vasil and Ochsner 1999; Yang et al. 2007). These iron uptake systems may also recognise synthetic iron-binding compounds, thus resulting in some chelators being able to stimulate growth rather than inhibiting growth. For example, it was shown (Visca et al. 2013) that DFP inhibits planktonic growth of *P. aeruginosa* at 0.92 mM (= 128 µg/mL) but stimulates its growth rate at low concentrations, and the authors thus appropriately described such chelators as having a “dual personality”.

Here we have found that the chelator CP94 can inhibit planktonic growth while promoting biofilm formation of *P. aeruginosa*. At high concentrations, it inhibits growth of planktonic cells, albeit that it is less toxic to *P. aeruginosa* than the closely related DFP. With respect to DFP, the MIC values determined in IMDM were similar to those determined in other studies (Richter et al. 2017; Thompson et al. 2012; Visca et al. 2013) even though these studies used different growth media. As mentioned above, the latter study also observed a stimulation of planktonic growth at low concentrations of DFP (11–22 µg/mL); we tested this at a similar range (8 and 16 µg/mL) but did not observe such a stimulation (data not shown). That, however, could be a consequence of the different growth medium used.

We also found that the inhibitory concentrations of CP94 and DFP were significantly higher in rich (LB) or iron-supplemented IMDM medium, with MIC values for both CP94 and DFP being fourfold-to eightfold higher, showing that the iron concentration is the main determinant in the antimicrobial activity of these chelators. Thus in this case, the mechanism of action of these chelators is through limiting the amount of free iron. Both DFP and CP94 are bidentate chelators that form a defined 3:1 complex with ferric iron and with comparable affinities as judged by pFe^3+^ values, a reliable measure of iron affinity at physiologically relevant pH (pFe^3+^_DFP_ = 20.6; pFe^3+^_CP94_ = 20.5) (Ma et al. 2013). Some antimicrobial activity was, however, retained even though the chelators were fully saturated with Fe^3+^. This may indicate that, at high concentrations (> 1024 µg/mL), these chelators do have an iron-independent activity as also suggested previously (Visca et al. 2013).

The most interesting observation from our study is that CP94 stimulates formation of *P. aeruginosa* biofilms, in particular at high concentrations that are inhibitory to planktonic growth. In contrast, DFP is inhibitory to biofilm formation, and it is conceivable that this is because DFP limits the amount of iron that is available to cells. For CP94, one possible explanation for this could be that this compound leads to a general stress response, which in some cases has been shown to induce biofilm formation (Harms et al. 2016; Poole 2012). However, this does not seem to be very likely as CP94 behaved very differently to its simple homologue DFP, and this effect was much more pronounced in cells lacking the endogenous siderophores, pyoverdine and pyochelin. Moreover, there was no stimulation of biofilm formation of two other Gram-negative bacteria (*E. coli* and *S.* Typhimurium), indicating that under the conditions tested, the stimulatory effect of CP94 is specific to *P. aeruginosa*. Another mechanism of biofilm stimulation has been observed with sub-inhibitory concentrations of the antibiotic tobramycin, which can increase biofilm formation by about twofold to threefold in both *P. aeruginosa* and *E. coli*. This stimulation is a consequence of a change in levels of cyclic di-guanosine monophosphate via an aminoglycoside-specific response regulator (Hoffman et al. 2005). It seems very unlikely that CP94 would affect Arr, and this is therefore another mechanism of action that can be excluded.

We did not test whether the increase in biofilm formation of *P. aeruginosa* was due to an increased number of cells, an increase in the amount of extracellular polymeric substance (EPS) in biofilms, or both. However, we did analyse the effects of CP94 on the early stages of biofilm formation, by direct counting of the number of cells adherent to polystyrene after 6 h of incubation. This showed that in the presence of CP94 there were significantly more adherent cells, suggesting that at least in part the effects observed after 24 h are also due to an increased number of biofilm cells.

Based on our observations, we hypothesised that CP94, but not DFP, can act as a siderophore when *P. aeruginosa* cells are in a biofilm state. We did note that 4 µM of Fe^3+^ alone can stimulate biofilm formation to a greater extent than CP94, and that addition of CP94 plus Fe^3+^ did not further of increase biofilm formation. However, at 4 µM it seems likely that the transport of Fe^3+^ is not a rate limiting step which is why addition of CP94 to a growth medium that has sufficient amounts of Fe^3+^ does not lead to a additional stimulation of biofilm formation. Therefore, to support our hypothesis that CP94 can transport Fe^3+^, we investigated whether CP94 could promote the uptake of other metals. We analysed Ga^3+^ and Cu^2+^, both of which have antimicrobial activity and can also prevent biofilm formation in *P. aeruginosa* (Harrison et al. 2008; Kaneko et al. 2007). Ga^3+^ was of particular of interest, as it has the same charge and size as Fe^3+^ (Chitambar 2016; Harris and Sephton 1977; Harris and Pecoraro 1983), yet is not redox-active resulting in toxic effects. Cu^2+^ was included as the antimicrobial activity of the Cu-DFP complex against *P.aeruginosa* has been reported (Leite et al. 2019). In this study we confirmed that Ga^3+^ and Cu^2+^ are toxic to *P. aeruginosa* and, importantly, that CP94 stimulates the antimicrobial activity of these metals only when this bacterium is growing in a biofilm. At the concentrations used, both Ga^3+^ and Cu^2+^, on their own, reduced biofilms by 20–30%, whereas this reduction was ~ 40–60% in the presence of CP94. The concentration of the latter was well below the MIC value and on its own did not have an effect on growth, and we therefore conclude that the increased antimicrobial activity of Ga^3+^ and Cu^2+^ could be the effect of CP94-mediated transport of these metals. This contrasted with DFP, which did not stimulate the antibiofilm activity of Ga^3+^, so here only CP94 appears to show a “Trojan Horse” effect in which compounds may carry a toxic metal into cells (Golonka et al. 2019; Mjos et al. 2016). This effect has been demonstrated before in *P. aeruginosa* with a complex of Ga^3+^ and the chelator DFO, which was shown to kill planktonic cells and inhibit the formation of biofilms (Banin et al. 2008).

Several other Ga^3+^ complexes have also been tested on various microbes, indicating the potential for Ga^3+^ complexes as antimicrobial agents (Kelson et al. 2013). Ga^3+^ compounds have been used clinically, for instance in PET imaging (Price and Orvig 2014) and gallium nitrate is an FDA-approved therapeutic agent for malignancy-associated hypercalcemia (Bernstein 1998; Chitambar 2012). Gallium nitrate also inhibits *P. aeruginosa,* with MIC values that are in the same range as achievable in cancer therapy, albeit that the antimicrobial activity of gallium nitrate was strain dependent (Bonchi et al. 2015). Notably, the therapeutic concentration of gallium nitrate for hypercalcemia of malignancy is 10–15 µM (Bernstein 1998). As shown here, 1 µM Ga^3+^ can inhibit biofilms by 40–60% when complexed with CP94, and one might thus speculate that concentrations that effectively inhibit *P. aeruginosa* biofilms are therapeutically achievable. However, it is at this stage of course not known whether the toxicity of the Ga-CP94 complex is different from gallium nitrate, and whether Ga-CP94 is also active against *P. aeruginosa* biofilms in vivo. CP94 has been tested as an enhancer of 5-aminolaevulinic acid induced photodynamic therapy and photo diagnosis in vitro and in vivo (Blake et al. 2011; Campbell et al. 2008). It also has been tested for treatment of iron overload, and its toxicity appears to be species-dependent; in rat, at a dose of 100 mg/kg, it effectively mobilised liver iron, but also showed some toxicity, while in guinea pig it lacked toxicity, but also lacked efficacy because of rapid inactivation (Porter et al. 1993). It is thus unclear whether CP94-Ga can be used as an anti-infective, and further tests are required for topical or systemic treatment of biofilm-related *P. aeruginosa* infections. With regards to copper, the concentration required to inhibit *P. aeruginosa* biofilms is considerably higher than for gallium, and it seems thus unlikely that effective doses of CP94-Cu are achievable considering the potential toxicity.

As shown here and in other studies, both copper and gallium have antimicrobial activity. For copper, its redox activity could be the reason for its antimicrobial properties, as it can act as a catalyst in Fenton and Haber–Weiss reactions, which result in the production of hydroxyl radicals that causes cellular damage (Grass et al. 2011). However, it has also been shown in *E. coli* there is no significant oxidative DNA damage in vitro (Macomber et al. 2007) and instead that there is non-redox dependent damage through the formation of Cu^1+^ ligands with sulfur clusters in dehydratases (Macomber and Imlay 2009). Bidentate chelators with a 3-hydroxypyridin-4-one structure such as DFP and CP94 form 2:1 complexes with Cu^2+^ (Leite et al. 2019), whereas in the case of Ga^3+^, DFP forms the expected 3:1 complex (Chaves et al. 2011) because not only it has the same charge as ferric ion, but also possesses a very similar ionic radius and shares the same preferred octahedral coordination geometry (Chitambar 2016; Harris and Sephton 1977; Harris and Pecoraro 1983). Therefore, Ga^3+^ is able to compete with Fe^3+^ to bind to iron-binding ligands in cells, such as heme and iron-sulfur cluster containing proteins. However, Ga^3+^ is, unlike Fe^3+^, not redox active and is not reduced to Ga^2+^ intracellularly, resulting in non-functional iron-dependent metabolic pathways which, in turn lead to bacterial cell death.

What remains still to be determined is how CP94 is taken up by *P. aeruginosa*. Generally, transport of iron in Gram-negative bacteria can follow a number of different routes. In *P. aeruginosa*, there are four such routes, being (a) uptake via siderophore complexes (in *P. aeruginosa* pyoverdine and pyochelin); (b) uptake via xenosiderophores, which are siderophores produced by other species; (c) uptake of heme from host heme proteins; and (d) uptake of ferrous iron (Cornelis and Dingemans 2013). The last route requires extracellular reduction of Fe^3+^ to Fe^2+^ and a dedicated FeoABC transporter (Cartron et al. 2006); this system is unlikely to be involved in what we observe here as, for instance, Ga^3+^ is not reduced under physiological conditions. The first three systems listed above all require specific TonB dependent receptors (TBDRs) in the outer membrane which are energised by a complex (TonB/ExbB/ExbD) in the cytoplasmic membrane, with transport across the cytoplasmic membrane being mediated by ABC transporters (Noinaj et al. 2010). For instance, ferric-pyoverdine is transported into the periplasm via the TBDRs FpvA and FpvB (Bodilis et al. 2009). In the periplasm, reduction of iron results in release from pyoverdine, and ferrous iron is then taken up through the ABC transporter FpvDE (Brillet et al. 2012). The route for ferripyochelin is somewhat different; the first step across the outer membrane is similar and involves the TonB-dependent receptor FptA (Ankenbauer and Quan 1994), but after this the entire ferripyochelin complex is taken up through the transporter FptX, and iron is released after reduction to Fe^2+^ in the cytoplasm (Youard et al. 2011). Interestingly, there are > 30 TonB-dependent receptors (Cornelis and Matthijs 2002), with some of these shown to be involved in the uptake of xenosiderophores (Cornelis and Dingemans 2013). Also, synthetic compounds may be taken up through such receptors and indeed, as mentioned before, DFP was shown to be taken up by planktonically growing *P. aeruginosa* cells at DFP concentrations that were below (20–160 µM; equivalent to 2.75–22 µg/mL) the MIC value (Visca et al. 2013). The receptor responsible for the uptake of DFP has not yet been identified but based on the results here it is likely to be a different receptor to the one responsible for the uptake of CP94, as the two compounds have opposite effects in our experiments, notwithstanding their very similar structures. We can also presume that this CP94 receptor is only expressed in cells that are growing as biofilms, as we did not observe the stimulatory effects on planktonic cells. Why the hypothetical CP94-receptor does transport DFP into cells is unclear at present. It is, however, interesting to note that CP94 is more lipophilic than DFP: the logD values for CP94 and its Fe^3+^ complex are 0.23 and − 0.62, whereas those for DFP and its Fe^3+^ complex are − 0.77 and − 2.60, respectively (Rai et al. 1998). So it could be that the hypothetical receptor has different affinity for the two compounds, but it is also conceivable that the difference in lipophilicity influences the diffusion of the two compounds through the biofilm matrix.

Although speculative, a number of potential transport routes can be considered for CP94. One of these is, for several reasons, the pyochelin transport route. Firstly, a proteome analysis showed that FptA, the receptor for pyochelin, is strongly overproduced in biofilms in a minimal medium containing 10 mM Ca^2+^, whereas there was no detectable production of FptA in either planktonic or biofilm cells in the absence of Ca^2+^ (Patrauchan et al. 2007). IMDM does contain 2 mM CaCl_2_, so it is conceivable that FptA is also overproduced in IMDM. It should however be noted that the medium used in that study also contains iron (0.36 µM), whereas IMDM does not have iron in its formulation, and it is unclear what effect this or other nutrients have on the levels of FptA in biofilms as compared to planktonic cultures. Secondly, the results from that proteomics study are corroborated by a search of the GEO database (Barrett et al. 2013), which showed that in LB medium, *fptA* was shown to be strongly upregulated (> sevenfold) in biofilms when compared to either 4-h or 24-h planktonically grown cells (GEO accession GSE30021, accessed May 2020; (Costaglioli et al. 2012)). Thirdly, a study showed that pyochelin was able to stimulate the uptake of Ga^3+^ in *P. aeruginosa*, via FptA and FptX, whereas this was not observed for other pyoverdine or chelators that make use of other transport routes (Frangipani et al. 2014). It should be noted that the structures of CP94 and pyochelin are quite different, and it is thus unclear whether FptA would be able to recognise CP94. However, another TBDR that should be considered is PiuA, which was shown to recognise and stimulate transport of hydroxypyridone-conjugated beta-lactams such as BAL30072 and MC-1 in *P. aeruginosa* PAO1 (Brown et al. 2013; Moynié et al. 2017). It is therefore conceivable that PiuA is capable of facilitating the transport of other structural analogues containing the hydroxypyridone moiety, which includes CP94. Interestingly, the aforementioned transcriptome study (Costaglioli et al. 2012) showed that, in addition to FptA, the only other TBDR that was overproduced in biofilms was PiuA, albeit that the increase was only 1.5 fold to twofold. However, we do again need to point out the differences in growth conditions used, and it is not yet known whether PiuA is overproduced in biofilms grown in IMDM. Nevertheless, CP94-metal complexes could potentially utilise a number of transport routes, with potential candidates those involving the TBDRs FptA and PiuA. In the future we aim to provide more direct evidence for the transport route used, by for instance investigating the effect of CP94 on biofilm formation of mutants of *fptA*, *piuA* or other genes encoding potential transporters.

Considering the activity of CP94-Ga and CP94-Cu on biofilms, it is also attractive to speculate on the clinical use of this complex as an antimicrobial agent since, as stated above, therapeutic concentrations of CP94-Ga^3+^ might be achievable. However, the applicability of CP94 complexes might also be somewhat limited as it is specific to biofilms and bacterial species that contain a CP94 uptake route. Our current research aims to explore the clinical applicability of CP94 further. Also of interest is to investigate whether CP94 could be used in combination with antibiotics, as several other chelator-antibiotic combinations have shown to have antimicrobial synergy (Chan et al. 2020; Lambert et al. 2004; van Asbeck et al. 1983b).

## Conclusion

In conclusion, the chelator CP94, but not the closely related DFP, acts as a siderophore for *P. aeruginosa* when the cells are in a biofilm state. This shows, as previously proposed (Visca et al. 2013), that chelators can have either inhibitory or stimulatory activity depending on various factors. In this case, the siderophore-like behaviour of CP94 is specific to *P. aeruginosa* as it does not appear to be taken up by other Gram-negative bacteria, and it is specific to cells in the biofilm state. Future studies in our laboratory are aimed at determining the transport route of CP94 as well as investigating the potential of CP94 as an anti-infective—either on its own or in combination with antibiotics.

## Supplementary information

Below is the link to the electronic supplementary material.

Supplementary material 1 (DOCX 162 kb)
