# Supplementary material for: Biofilm-specific uptake of a 4-pyridone-based iron chelator by Pseudomonas aeruginosa
Source: Biometals. 2021 Jan 11;34(2):315–28. doi: 10.1007/s10534-020-00281-x (PMC7940164; doi:10.1007/s10534-020-00281-x)
Supplement: Supplementary file 1 — Supplementary material 1 (DOCX 162 kb) [file 10534_2020_281_MOESM1_ESM.docx]

**Supplementary data**

**
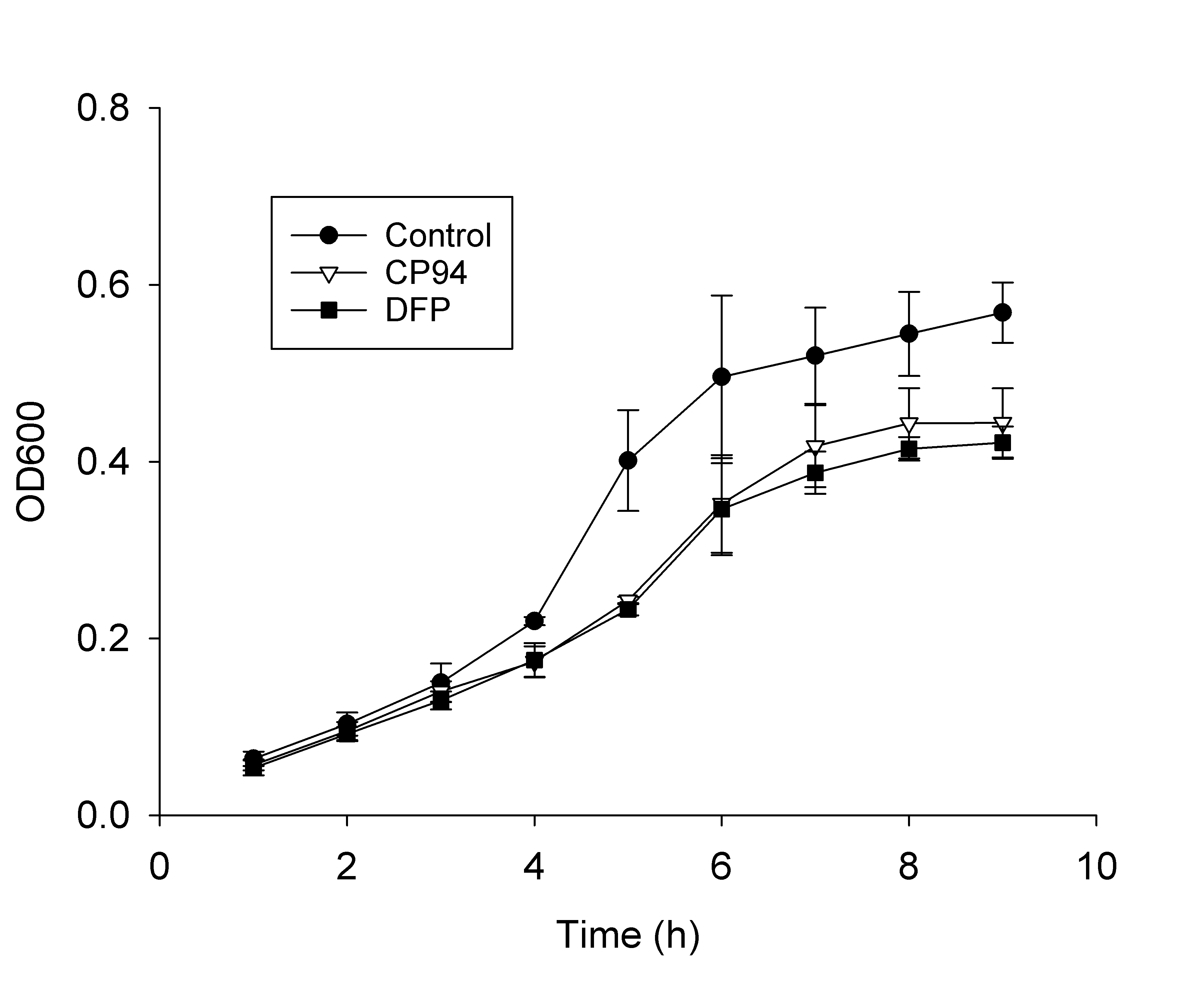
**

Supplementary Fig 1. Growth curves of *P. aeruginosa* PAO1 in IMDM, in the presence of subinhibitory concentrations of CP94 or DFP (64 µg/mL).

Supplementary Fig 2. Growth curves of PAO1 (A) and IST27 (B) in IMDM, in the absence of compounds (control), 12 µM CP94, 1 µM Ga^3+^, or both CP94 and Ga^3+^. Results are expressed as means +/- the SD (n=3).

Supplementary Fig 3. Activity of DFP saturated with Ga^3+^ against biofilms of *P. aeruginosa* PAO1 (A), IST27 (B) and ΔΔPAO1 (C). Error bars indicate standard deviation of 3 independent experiments. Statistical analysis was done using a 1-way ANOVA followed by a Tukey’s posthoc test to pairwise compare the means of the different treatments. None of the data showed a statistically significant difference.
